# Comparison of GRACE and TIMI risk scores in the prediction of in-hospital and long-term outcomes among East Asian non-ST-elevation myocardial infarction patients

**DOI:** 10.1186/s12872-021-02311-z

**Published:** 2022-01-07

**Authors:** Lu Yanqiao, Lan Shen, Miao Yutong, Shen Linghong, He Ben

**Affiliations:** 1 Department of Cardiology, Shanghai Chest Hospital, Shanghai Jiao Tong University, Shanghai, China; 2 Clinical Research Center, Shanghai Chest Hospital, Shanghai, China

**Keywords:** NSTEMI, GRACE, TIMI, Outcome assessment

## Abstract

**Background:**

Risk stratification in non-ST segment elevation myocardial infarction (NSTEMI) determines the intervention time. Limited study compared two risk scores, the Thrombolysis in Myocardial Infarction (TIMI) and Global Registry of Acute Coronary Events (GRACE) risk scores in the current East Asian NSTEMI patients.

**Methods:**

This retrospective observational study consecutively collected patients in a large academic hospital between 01/01 and 11/01/2017 and followed for 4 years. Patients were scored by TIMI and GRACE scores on hospital admission. In-hospital endpoints were defined as the in-hospital composite event, including mortality, re-infarction, heart failure, stroke, cardiac shock, or resuscitation. Long-term outcomes were all-cause mortality and cardiac mortality in 4-year follow-up.

**Results:**

A total of 232 patients were included (female 29.7%, median age 67 years), with a median follow-up of 3.7 years. GRACE score grouped most patients (45.7%) into high risk, while TIMI grouped the majority (61.2%) into medium risk. Further subgrouping the TIMI medium group showed that half (53.5%) of the TIMI medium risk population was GRACE high risk (≥ 140). Compared to TIMI medium group + GRACE < 140 subgroup, the TIMI medium + GRACE high-risk (≥ 140) subgroup had a significantly higher in-hospital events (39.5% vs. 9.1%, *p* < 0.05), long-term all-cause mortality (22.2% vs. 0% *p* < 0.001) and cardiac death (11.1% vs. 0% *p* = 0.045) in 4-year follow-up. GRACE risk scores showed a better predictive ability than TIMI risk scores both for in-hospital and long-term outcomes. (AUC of GRACE vs. TIMI, In-hospital: 0.82 vs. 0.62; long-term mortality: 0.89 vs. 0.68; long-term cardiac mortality: 0.91 vs. 0.67, all *p* < 0.05). Combined use of the two risk scores reserved both the convenience of scoring and the predictive accuracy.

**Conclusion:**

GRACE showed better predictive accuracy than TIMI in East Asian NSTEMI patients in both in-hospital and long-term outcomes. The sequential use of TIMI and GRACE scores provide an easy and promising discriminative tool in predicting outcomes in NSTEMI East Asian patients.

**Supplementary Information:**

The online version contains supplementary material available at 10.1186/s12872-021-02311-z.

## Background

Cardiovascular disease remains the main contributor to the cause of death globally [1, 2]. The prevalence of acute coronary syndromes (ACS) has increased significantly during the recent 30 years in China [2, 3]. A major component of ACS, Non-ST segment elevation myocardial infarction (NSTEMI), has more than twice the incidence compared to ST-segment elevation myocardial infarction (STEMI) [4, 5]. However, NSTEMI patients have a large range of clinical consequences, from minimal sequelae to early death [6]. Risk assessment was crucial in guiding therapeutic decision-making. Guidelines recommend that high-risk patients receive more aggressive invasive treatment upon risk stratification on admission [7–9]. Therefore, there is an essential need to assess individual risk easily and accurately. GRACE and TIMI are the two most popular prediction models in ACS developed during the prereperfusion era and have been compared face to face mainly in the Caucasian population [10, 11]. Studies proved TIMI’s advantage in its simplicity in clinical application and GRACE's favorable discriminative power [12, 13]. Nevertheless, limited evidence compared the usefulness of these two major risk scores and their association with both in-hospital events and long-term outcomes in contemporary East Asian patients.

In this observational study, we sought to (1) examine the distribution of risk tertiles by GRACE versus TIMI in a contemporary cohort of NSTEMI East Asian patients in today's reperfusion era, (2) compare the predictive ability between GRACE and TIMI risk score, (3) explore the optimal way for risk stratification in East Asian population by combining these two scores.

## Methods

### Study population

The study population began with 1659 patients consecutively hospitalized with primary diagnose as ACS in the cardiovascular department in Shanghai Renji hospital, China, from Jan 1st, 2017 to Nov 1st, 2017. Participants were at least 18 years old, admitted to the hospital with a presumptive ACS diagnosis according to the following points: clinical symptoms, typical ischemic ECG changes, elevated cardiac biomarkers, and documented prior coronary artery diseases [14]. Participants diagnosed with STEMI were excluded. Patients were also excluded if the symptom was caused by severe trauma, medical surgery, or other serious comorbidities. Briefly, we included NSTEMI patients with acute chest discomfort, elevation of cardiomyocyte necrosis biomarkers, ECG characteristics (transient ST-segment elevation, persistent or transient ST-segment depression, flat or inverted T waves, pseudo-normalization of T waves, or normal ECG) [14].

### Risk scores

Both GRACE and TIMI risk scores were calculated on admission. In UA/NSTEMI, TIMI scores incorporated the following 7 points: age ≥ 65 years, ≥ three coronary artery disease (CAD) risk factors, known coronary stenosis > 50%, aspirin used in the past 7 days, severe angina (≥ 2 episodes w/in 24 h), severe angina (≥ 2 episodes w/in 24 h) and positive cardiac marker. Patients achieved 1 point for yes and 0 points for no. Patients with 0–2 scores were low-risk groups while 3–4 scores were medium-risk groups and 5–7 scores were high-risk.

GRACE score was calculated on admission based on the following variables: age, heart rate (HR), systolic blood pressure (SBP), initial serum creatine (Cr), Killip classification, cardiac arrest at presentation, initial cardiac enzyme positive, and ST-segment deviation. Patients who scored ≤ 108 points were low-risk, 109–139 points were medium-risk, ≥ 140 points were high-risk. We also used online risk calculators following the recommendation by 2020 ESC guideline, https://www.outcomes-umassmed.org/risk_models_grace_orig.aspx [14].

To explore the optimal risk score in our study population, we examine a way to reserve the two risk scores’ convenience and predictive accuracy. We tested the method to use the combined score, which further stratified TIMI medium-risk patients into the GRACE ≥ 140 subgroup and GRACE < 140 subgroups.

### Outcomes

The primary outcomes of interest for the present study were the following: (1) in-hospital composite endpoint including all-cause mortality, re-infarction, heart failure, cardiac shock, resuscitation, and in-hospital stroke, (2) long-term outcomes were all-cause mortality and cardiac mortality from discharge to 4-year follow-up post-discharge. Long-term all-cause death information was collected through two independent clinical follow-up fellows by telephone or email.

### Statistical analysis

Continuous variables were presented using medians with interquartile ranges (IQRs) and were compared using Wilcoxon rank-sum tests. Categorical variables were presented as percentages and compared using Mantel–Haenszel χ^2^ tests. The accuracy of predicting in-hospital and long-term outcomes between TIMI and GRACE score was evaluated by the Receiver Operating Characteristic Curve (ROC) curves. Discriminatory performance was measured by the c-statistic (area under the receiver-operating characteristic curve) comparing the TIMI risk score with the GRACE risk score. Kappa value was calculated to analyze the consistency of TIMI and GRACE risk scores. Kappa value < 0.4 was considered an inconsistency between the two comparisons. SPSS version 21.0 (IBM) was used to analyze all the statistical data, and a two-sided *p* value < 0.05 was considered statistically significant.

## Results

**Fig. 1:**
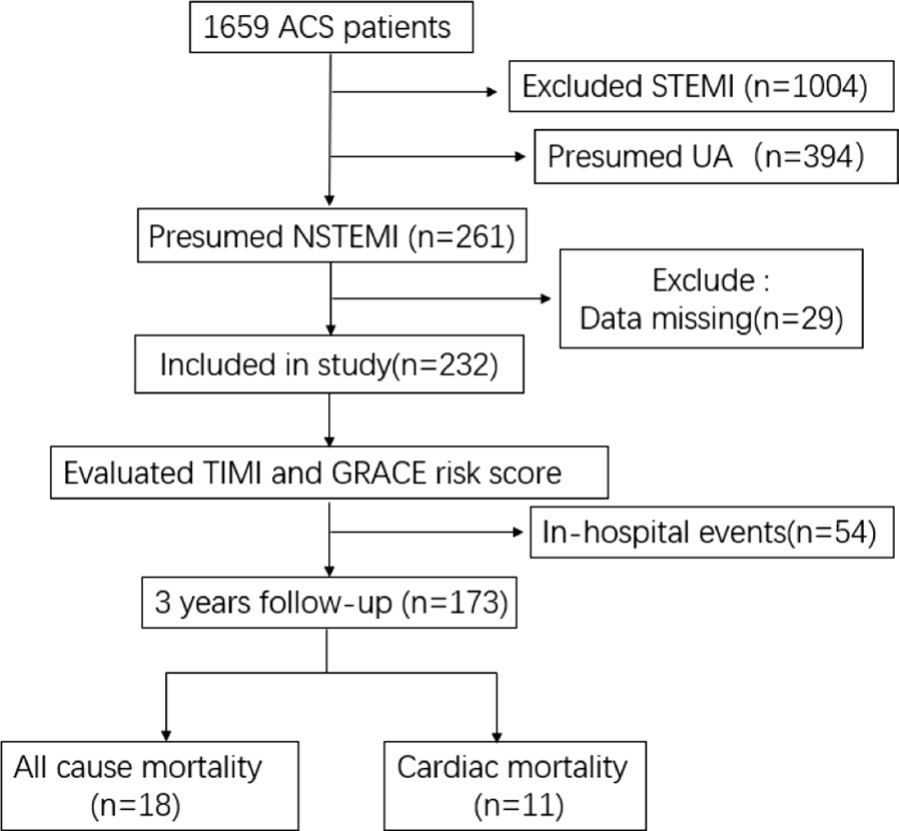
Patients flow chat and occurrence of in-hospital events. *STEMI* ST segment elevation myocardial infarction, *NSTEMI* non ST segment elevation myocardial infarction

Of 1659 ACS patients screened for the study, 261 patients were hospitalized as NSTEMI with positive troponin test. Among these NSTEMI patients, 29 participants were excluded with missing clinical data, leaving 232 participants were eligible as NSTEMI patients in our study (Fig. 1).

Among the NSTEMI patients, 163 (70.3%) patients were male, while 69 (29.7%) patients were female. In the 232 patients, 224 (96.6%) underwent coronary angiography, and 166 (74.1%) patients underwent revascularization. More than half (65.9%) of patients suffered from hypertension, 32.8% had a history of diabetes, 5.2% had hyperlipidemia, and 38.8% had a smoking history. One in five had a history of myocardial infarction, and 18.1% had prior PCI history (Table 1).

**Table 1:** Baseline characteristics of patients with NSTEMI

Variables	Patients (n = 232)	With 4-year followup (n = 173)	Without 4-year follow-up (n = 59)	*p* value*
*Admission baseline*				
Sex (male: female)	70.3%: 29.7%	71.1%: 28.9%	67.8%: 32.2%	0.743
Age (years)	67.0 (59.0–74.0)	67.0 (59.5–74.0)	68.0 (58.0–76.0)	0.721
HR (beats/min)	76.0 (70.0–82.0)	75.0 (66.3–80.0)	78.0 (70.0–82.0)	0.623
DBP (mmHg)	77.0 (70.0–85.0)	77.0 (70.5–85.0)	76.0 (70.0–82.0)	0.960
SBP (mmHg)	137.5 (125.0–154.0)	139.0 (125.0–154.5)	136.0 (125.0–153.0)	0.726
GLU (mmol/L)	5.6 (4.8–7.2)	5.5 (4.8–6.6)	5.8(4.9–8.6)	0.163
Hb (g/L)	135.0 (120.8–147.3)	136.0 (121.3–147.0)	132.0 (117.0–148.3)	0.605
PLT (*10^9^/L)	202.0 (166.5–246.5)	201.0 (167.5–242.0)	208.0 (163.3–258.8)	0.619
APTT (s)	27.6 (25.7–29.9)	27.5 (25.7–29.8)	27.9 (25.6–31.5)	0.709
SCr (μmmol/L)	77.0 (64.9–93.0)	77.0 (64.5–92.0)	75.3 (64.8–101.1)	0.536
LVEF (%)	60.0 (55.0–66.0)	61.0 (55.0–67.0)	59.5 (55.0–64.0)	0.439
LVEDV/BSA (ml)	47.0 (43.0–50.0)	46.5 (43.0–51.0)	47.5 (42.3–50.0)	0.543
CKMB (IU/L)	20.3 (13.5–34.5)	21.3 (13.6–38.1)	18.3 (12.8–31.1)	0.272
Tn I (ng/ml)	1.0 (0.2–5.1)	1.3 (0.2–5.7)	0.9 (0.2–2.9)	0.384
CK (U/L)	128.0 (79.0–321.5)	134.0 (78.0–340.0)	113.0 (79.0–305)	0.619
Peak TNI (ng/ml)	9.45 (2.3–18.2)	8.73 (3.5–15.3)	10.21 (2.2–16.2)	0.732
Peak CK (ng/ml)	203.0 (68.0–340.0)	225.0 (89.0–354.0)	194.0 (65.0–332.0)	0.630
LDL-C (mmol/L)	2.6 (1.9–3.1)	2.6 (1.8–3.1)	2.7 (2.0–3.5)	0.276
HDL-C (mmol/L)	1.0 (0.8–1.2)	1.0 (0.8–1.2)	1.0 (0.8–1.2)	0.743
TG (mmol/L)	1.6 (1.1–2.3)	1.5 (1.2–2.2)	1.8 (1.1–2.4)	0.302
CRP (mg/L)	3.2 (1.0–9.0)	12.8 (1.0–8.6)	4.4 (1.0–9.5)	0.619
BNP (pg/mL)	136.0 (61.5–327.5)	114.0 (58.0–316.0)	146.5 (89.6–345.0)	0.131
*Previous disease (%)*				
Hypertension	65.9	67.6	61.0	0.427
Hyperlipidemia	5.2	5.2	5.1	1.000
Diabetes	32.8	30.1	40.7	0.150
Smoking	38.8	38.7	39.0	1.000
Ischemia stroke	13.4	11.0	20.3	0.068
Renal insufficiency	8.6	9.2	6.8	0.609
Angina	40.9	42.2	37.3	0.543
Prior heart failure	3.9	4.0	3.4	1.000
Prior MI	19.0	18.5	20.3	0.848
PCI	18.1	16.8	22.0	0.433
CABG Killip classes	2.6	3.5	0	0.342
I	79.3	79.2	79.7	0.939
II	4.7	4.6	5.1	1.000
III	9.5	9.2	10.2	0.835
IV	6.5	6.9	5.1	0.847
Cardiac arrest at admission	0.4	–	–	–
*Discharge medication (%)*				
Statins	91.8	90.2	96.6	0.200
ACE inhibitors or ARBs	46.1	46.8	44.1	0.714
β-blockers	64.2	67.6	54.2	0.064
*Risk scores*				
GRACE scores	137.0 (114.3–157.8)	137.0 (114.5–157.0)	140.0 (113.0–164.0)	0.764
TIMI scores	3.0 (2.0–4.0)	3.0 (2.0–4.0)	3.0 (2.0–4.0)	0.564

Values are showed as median (interquartile range), or percentage (n%)

*HR* heart rate, *DBP* diastolic blood pressure, *SBP* systolic blood pressure, *GLU* blood glucose, *TG* triglyceride, *Hb* hemoglobin, *PLT* platelet, *APTT* activated partial thromboplastin time, *CKMB* creatine phosphokinase-Mb, *SCr* serum creatinine, *CK* creatine kinase, *CRP* C-reactive protein, *BNP* brain natriuretic peptide, *LEVF* left ventricular ejection fraction, *LVEDV/BSA* left ventricular end-diastolic volume/body surface area, *MI* myocardial infarction

*p < 0.05

**Fig. 2:**
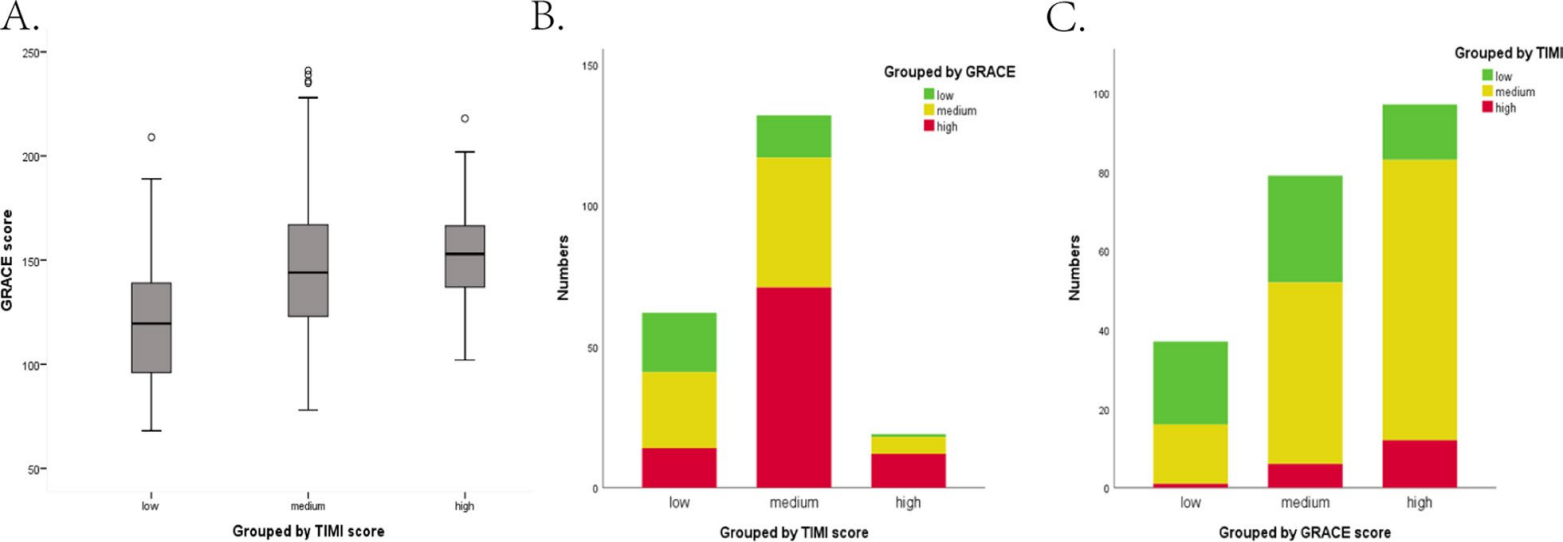
The correlation between TIMI and GRACE risk scores showed on the scatterplot and boxplot. **A** The boxplot showed the mean GRACE score (Y axis) in different TIMI risk groups (X axis). **B** The bar graph showed the proportion of different GRACE risk grades (Y axis) in different TIMI risk groups (X axis). **C** The bar graph showed the proportion of different TIMI risk grades (Y axis) in different GRACE risk groups (X axis)

TIMI and GRACE risk scores were evaluated for every patient on admission. The median TIMI risk score was 3.0 (2.0–4.0), and the median GRACE score was 137.0 (114.3–157.8). These 232 patients were divided into three groups (high-risk group, medium-risk group, and low-risk group) based on their risk scores. There was a large discordance between these two risk scores. GRACE grouped most people into high-risk (45.7%) while TIMI grouped the majority into medium-risk (61.2%) (Additional file 1: Table S1). Half of the TIMI medium-risk group (53.5%) were GRACE high-risk patients. Similarly, the majority of the TIMI low-risk group was GRACE medium-risk patients (43.5%). In contrast, the majority of GRACE high-risk patients (71.7%) were TIMI medium risk. There is little correlation between GRACE and TIMI risk score. (Kappa value = 0.077, Additional file 1: Table S1 and Fig. 2).

In-hospital events in patients grouped by GRACE score were 38.7% in GRACE high, 11.8% in GRACE medium, and 7.3% in GRACE low-risk groups (Table 2). In-hospital events grouped by TIMI score were 38.1% in TIMI high, 25.4% in TIMI medium, and 14.5% in TIMI low-risk patients (Table 2). Notably, the TIMI medium and GRACE medium showed a significant difference in the incidence of in-hospital events (25.4% vs. 11.8%, *p* = 0.014, Table 2). When we mixed the medium-risk group with the high-risk group patients, the prediction difference of TIMI and GRACE risk scores disappeared (Additional file 2: Table S2).

**Table 2:** Occurrence of in-hospital events and long-term outcomes in patients grouped by TIMI and GRACE risk scores

	GRACE risk score	TIMI risk score	*p* value
In-hospital events			
Low risk	3 (41)	10 (69)	0.411
Medium risk	10 (85)	36 (142)	0.014*
High risk	41 (106)	8 (21)	0.960
All-cause mortality			
Low risk	0 (29)	3 (52)	0.549
Medium risk	0 (68)	12 (105)	0.01*
High risk	18 (76)	3 (16)	0.921
Cardiac mortality			
Low risk	0 (29)	2 (52)	0.535
Medium risk	0 (67)	6 (104)	0.082
High risk	11 (76)	3 (16)	0.960

Results are showed as value (n). *p < 0.05

**Fig. 3:**
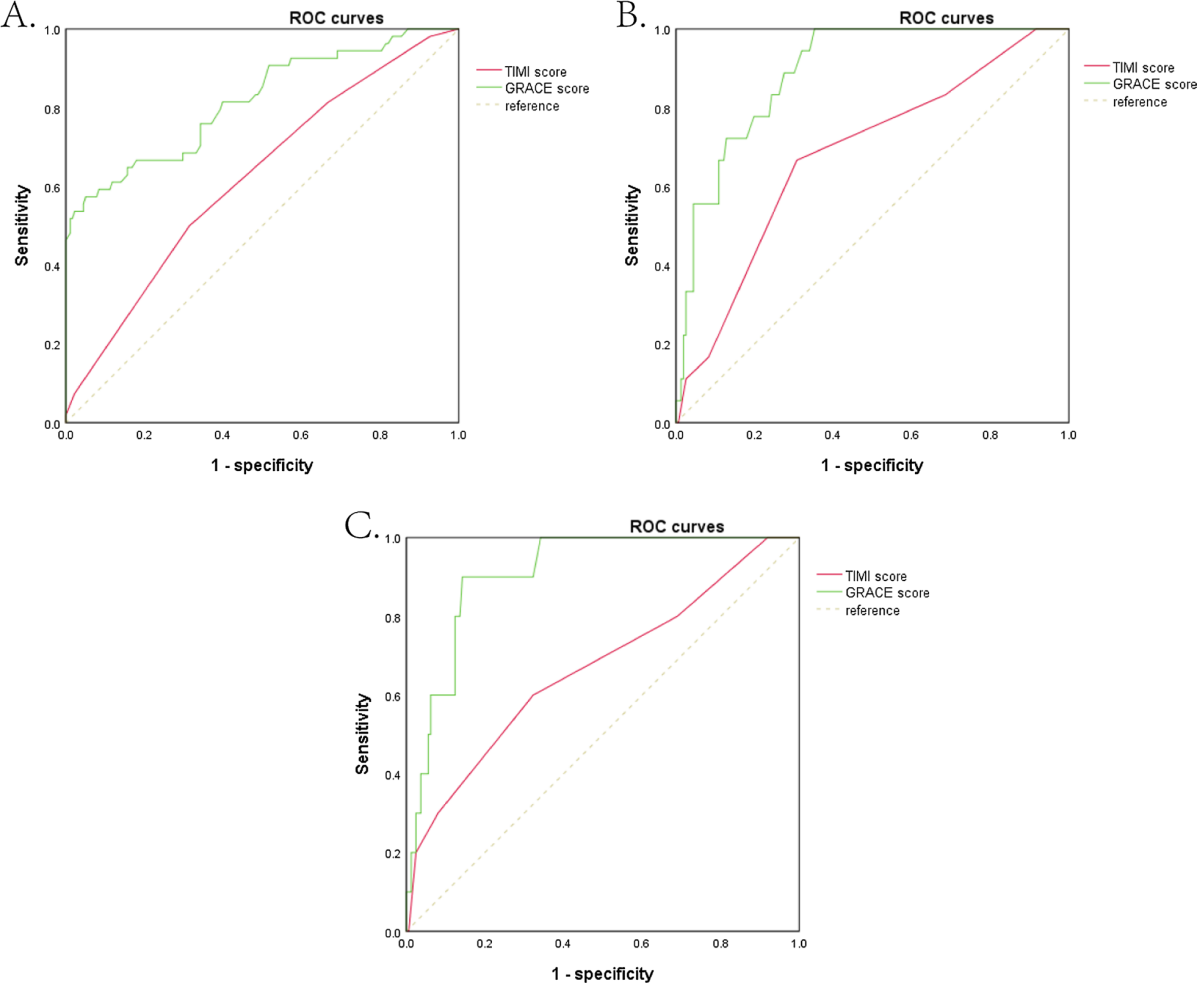
Discrimination of GRACE and TIMI scores for prediction of in-hospital and long-term outcomes. **A**. ROC curve of TIMI score and GRACE score in predicting in-hospital events. AUC of GRACE score and TIMI score under ROC curve: 0.82 (95% CI 0.75–0.89) versus 0.62 (95% CI 0.54–0.71), *p* < 0.001. **B** ROC curve of TIMI score and GRACE score in predicting all-cause mortality. AUC of GRACE score and TIMI score under ROC curve: 0.89 (95% CI 0.83–0.95) versus 0.68 (95% CI 0.55–0.81), *p* = 0.002. **C** ROC curve of TIMI and GRACE score in predicting cardiac mortality. AUC of GRACE score and TIMI score under ROC curve: 0.91 (95% CI 0.84–0.97) versus 0.67 (95% CI 0.48–0.6), *p* = 0.006

We used ROC curves to evaluate the accuracy of predicting in-hospital events with GRACE and TIMI risk scores. As shown in Fig. 3, both GRACE and TIMI risk scores had predictive value for the occurrence of in-hospital events. However, GRACE risk scores showed better predictive ability than TIMI risk scores (C-statistic for GRACE versus TIMI: 0.82, 95% CI 0.75–0.89 vs. 0.62, 95% CI 0.54 to 0.71, *p* < 0.001).

During the 4-year follow-up, 59 of them lost to follow-up. We compared patients who had follow-up and those who lost follow-up. The baseline characteristics were similar between the two groups (Table 1).

During the 4-year follow-up (median follow-up time was 3.7 years, the interquartile range was 3.4–3.9 years), 18 all-cause deaths were observed, 11 of them were cardiac mortality (Fig. 1). For GRACE grouping, all death occurred in GRACE high-risk group (Table 2). For TIMI grouping, the high, medium, and low-risk group had a mortality rate of 18.8%, 11.4%, and 5.8%, respectively (Table 2). The cardiac mortality rate in GRACE high-risk group was 14.5%. In the TIMI high, medium, low-risk group, the cardiac mortality incidence was 18.8%, 5.8%, 3.8%. GRACE score showed higher prediction ability than TIMI score in all-cause mortality and cardiac mortality (AUC under ROC curves: 0.89 vs. 0.68, 0.91 vs. 0.67, Fig. 3).

Similarly, the incidence of long-term outcomes was significantly different between TIMI medium-risk group and GRACE medium-risk group (Table 2). However, when we combined the medium and high-risk groups, the predictive difference disappeared between TIMI and GRACE risk scores (Additional file 2: Table S2).

**Fig. 4:**
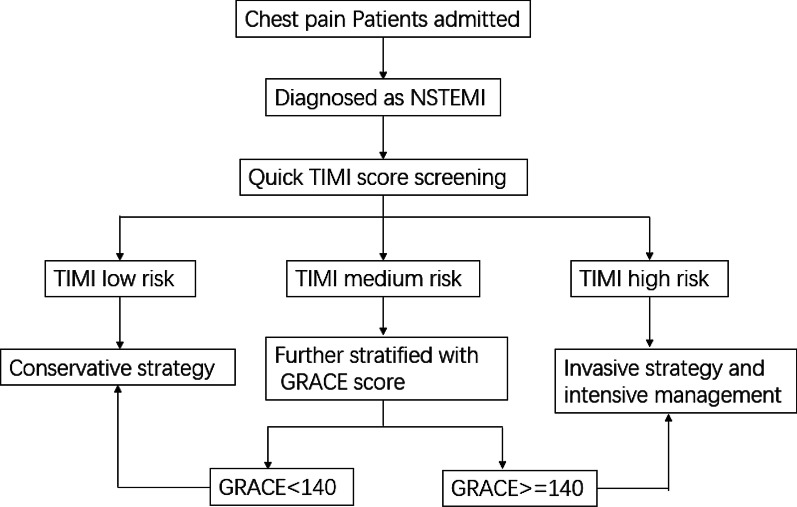
Flow chat of screening high-risk patients with combination TIMI and GRACE risk score. *NSTEMI* non-ST-segment elevation myocardial infarction

Due to significant heterogeneity in TIMI medium-risk groups, we further selected out high-risk patients among TIMI medium-risk groups by subsequently using the GRACE score. We first apply the TIMI score for initial screening. If patients were TIMI low or TIMI high, we stopped. If patients were TIMI medium, we used the GRACE score for further subgrouping (Fig. 4). Compared with the TIMI medium + GRACE high risk (< 140) subgroup, patients in TIMI medium + GRACE ≥ 140 subgroup were older, had higher brain natriuretic peptide (BNP), and were more likely to have comorbidities such as a history of stroke and heart failure. (Additional file 3: Table S3) Among the TIMI medium group, the GRACE ≥ 140 subgroup had an almost six-fold increased risk of in-hospital death than the GRACE < 140 subgroup (39.5% vs. 9.1%, odds ratio: 6.52, 95% CI 2.5–17.0, *p* < 0.001).

Among the 142 TIMI medium-risk patients, 76 were sub-grouped as GRACE high (≥ 140), and 54 were followed up for 4 years. (Table 3) Similar to in-hospital events, the TIMI medium + GRACE high (≥ 140) subgroup had higher all-cause mortality and cardiac death. (all-cause mortality: 22.2% vs. 0% *p* < 0.001, cardiac death:11.1% vs. 0% *p* = 0.045, Table 3). In short, TIMI medium-risk group had a widely divergent trend in event risk. The TIMI medium + GRACE high (≥ 140) subgroup has a similar event risk to the TIMI high-risk group, whereas the TIMI medium + GRACE < 140 subgroup has a similar event risk to the TIMI low-risk group.

**Table 3:** The risk heterogeneity of in-hospital events and long-term outcomes in TIMI medium risk group

	GRACE high subgroup	GRACE non-high subgroup	*p* value
In-hospital events	30/76, 39.5%	6/66, 9.1%	< 0.001
All-cause mortality	12/54, 22.2%	0/51, 0%	< 0.001
Cardiac mortality	6/54, 11.1%	0/50, 0%	0.045

Results are showed as value/n, percentage (n%)

## Discussion

In today's reperfusion era, risk score decides the optimal timing of intervention among NSTEMI, yet the optimal invasive time and specific long-term management by risk stratification are still upon disputation [15–17]. Moreover, the significant difference in patient characteristics between Caucasian and Asian NSTEMI patients further highlights the need to examine TIMI and GRACE risk scores in East Asian NSTEMI patients [18]. To accurately assess the discriminative power of GRACE and TIMI risk score among the East Asian NSTEMI population, our study focused on the NSTEMI patients instead of the ACS group, who most likely are in urgent need of aggressive invasive therapy upon risk stratification. As the first study, we examined the in-hospital and 4-year long-term outcomes’ predictive value of these two major risk scores in current clinical practice.

The baseline characteristics of East Asian populations were largely different from the Caucasian population. Compared to the original GRACE cohort [19], our population was less likely to have a history of hyperlipidemia, angina, myocardial infarction, CABG, and heart failure but more likely to have ischemia, stroke, hypertension, diabetes, and renal insufficiency. We observed a significantly low incident of hyperlipidemia (5.2% vs. 43.6%) in our population, which may be due to the difference in races and lifestyles between East Asians and Caucasians. Similarly, one recent large Korea registry of 27,852 acute myocardial infarction patients showed that the dyslipidemia rate in Korea was 9.5%, compared with 53% in the Europe and America cohort [20]. Our study found that GRACE has higher discriminatory power in predicting in-hospital events and long-term outcomes than TIMI in East Asian NSTEMI patients. Such finding echoes previous studies examining these two scores' prediction ability among different ethnic NST-ACS/NSTEMI groups (such as UK, Portugal, and Latin-American population) [21–23]. A recent study on NSTEMI reported that the GRACE score predicted 28-day mortality better than the TIMI score (AUC: 0.87 vs. 0.54) [24]. Another study analyzed the 2184 NSTEMI patients in Korea, found that the AUC of ROC curves of GRACE and TIMI risk score is 0.750 versus 0.616 [25]. Until now, limited research studied the 4-year long-term outcomes between these two risk scores in the East Asian population. None of these studies tried to figure out the exact predictive discordance between TIMI and GRACE risk scores.

We found that GRACE stratified most patients into high-risk while TIMI stratified the majority as medium-risk. Half of the TIMI medium group are GRACE high-risk patients with similar in-hospital events and long-term outcomes risk as TIMI high, mirroring the prior observed observations that TIMI tends to stratify high-risk patients into medium risk [13]. That may minimize doctors’ attention to these patients. Previous studies in the Caucasian population were consistent with this result. A Britain study that examined 104 Non-ST-Elevation Acute Coronary Syndrome (NSTE-ACS) patients found that most patients were classified as a medium risk by TIMI score (64%), yet classified as high risk with another risk stratification score—CMNW (Cheshire, Merseyside and North Wales Cardiac Network) score (60%) [22]. Combining the TIMI and GRACE scores improves the predictive value, reserving the convenience of scoring while improving its diagnostic accuracy. To the best of our knowledge, the present study is the first to examine the discrepancy between risk scores in detail and explore the optimal risk stratification strategy among East Asian NSTEMI in both in-hospital and long-term outcomes.

Several findings can explain this discordance among risk scores. First, cardiac biomarker elevation only got 1 point in TIMI, which had a low weight in the TIMI scoring system but a high GRACE score. Therefore, although all our study population was troponin positive with a median GRACE score of 140 (135–145), approximately 30% of patients were still in TIMI low-risk groups (≤ 2 points). Second, the TIMI score variables did not comprehensively summarize high-risk patients' characteristics, such as heart rate, blood pressure, cardiac arrest, and ST-deviation, included in the GRACE score system. Therefore, the TIMI score underestimated patients' risk was particularly-risk patients. Those high-risk patients labeled as TIMI medium would be underestimated their risk of in-hospital events, delayed prompt invasive therapy, and subject to increase long-term mortality.

In conclusion, an optimal risk score requires the convenience of utility and the accuracy of discrimination. The GRACE risk score’s main limitation is its apparent “complexity”, requiring specific calculator tools for its use at the bedside. In contrast, TIMI was regarded as a risk score that is simpler to use and widely applicable in the emergency room than other risk scores [26]. Physicians may be reluctant to use risk scores at the bedside when they find it inconvenient and time-consuming. Moreover, most patients were decided with invasive therapy in the Emergency room. Thus, combine the two risk scores reserving the convenience of scoring while improving its diagnostic accuracy. The combination reaches a similar discriminative power as the GRACE score and preserves the TIMI score’s easy use, meaning more patients can be easily scored in the emergency room, thereby improving patient care in routine clinical practice.

## Limitations

The study was a retrospective study from Jan 1st, 2017, to Nov 1st, 2017. Although we collected the data consecutively, participants included in this study were from a single center that may lack representation which might cause recruitment deviation. Although their baseline characteristics did not differ from the published largest Chinese ACS patients' cohort [27], we could not eliminate any potential bias. Second, our study population is not as large as many registry studies. Although it is one of the largest NSTEMI East Asian population studies, our findings may not apply to patients who differ significantly from our population. Finally, 4 years after discharge, about 25% of patients lost to follow-up. Since our study was real-world data, the percentage of long-term follow-up in real-world populations was similar to previous studies [28, 29]. Moreover, our studies compared the patients' characteristics between those with and without 4-year follow-up, showing no difference in patients' baseline characteristics and medical treatment. Therefore, the loss of follow-up should not skew our result very much.

## Conclusion

In this retrospective study with East Asian NSTEMI patients, GRACE scores better-predicted patient’s in-hospital events and long-term outcomes than TIMI scores. Physicians can use the TIMI score for first screening and further categorize the medium-risk group by the GRACE score, a novel way to facilitate risk score use and improve the identification of high-risk patients.

## Supplementary Information

**Additional file 1**. **Table S1** showed the percentage of patients in different risk groups divided by TIMI and GRACE risk score. Kappa value showed the discordance of TIMI and GRACE risk score in grouping different-risk-patients. (Kappa value < 0.4 implied that the correlation between TIMI and GRACE was poor.).

**Additional file 2**. **Table S2** showed that after mixing medium- risk group and high-risk group in TIMI and GRACE risk groups, the incidence of in-hospital events and long-term outcomes showed no different. Results were showed as value (n).

**Additional file 3**. **Table S3** showed the difference of baseline characteristics between patients with a high GRACE score and those with a low GRACE score in the TIMI medium-risk group. Results were showed as median (interquartile range), or percentage (n%).

## Data Availability

The data set that supports the findings of this study are available from the corresponding author (drheben@126.com), upon reasonable request. The supplementary data is available online.

## Abbreviations

NSTEMI: Non-ST segment elevation myocardial infarction
TIMI: Thrombolysis in myocardial infarction
GRACE: Global registry of acute coronary events
ACS: Acute coronary syndromes
STEMI: ST-segment elevation myocardial infarction
HR: Heart rate
SBP: Systolic blood pressure
Cr: Creatine
ROC: Receiver Operating Characteristic Curve
BNP: Brain natriuretic peptide
