# Supplementary material for: Comparison of GRACE and TIMI risk scores in the prediction of in-hospital and long-term outcomes among East Asian non-ST-elevation myocardial infarction patients
Source: BMC Cardiovasc Disord. 2022 Jan 7;22:4. doi: 10.1186/s12872-021-02311-z (PMC8742311; doi:10.1186/s12872-021-02311-z)
Supplement: Supplementary file 1 — Additional file 1. Table S1 showed the percentage of patients in different risk groups divided by TIMI and GRACE risk score. Kappa value showed the discordance of TIMI and GRACE risk score in grouping different-risk-patients. (Kappa value < 0.4 implied that the correlation between TIMI and GRACE was poor.). [file 12872_2021_2311_MOESM1_ESM.docx]

**Table S1. The contrast between patients grouped with GRACE risk score and patients grouped with TIMI risk score.**

|  | GRACE  n=232 | TIMI  N=232 | P value | Kappa value |
| --- | --- | --- | --- | --- |
| Low-risk | 17.7% | 29.7% | 0.002 | 0.077 |
| Medium-risk | 36.6% | 61.2% | <0.001 |  |
| High-risk | 45.7% | 9.1% | <0.001 |  |

Kappa value- the discordance of TIMI and GRACE risk score in grouping different-risk-patients. (Kappa value < 0.4 implied that the correlation between TIMI and GRACE was poor.)
