# Supplementary material for: Comparison of GRACE and TIMI risk scores in the prediction of in-hospital and long-term outcomes among East Asian non-ST-elevation myocardial infarction patients
Source: BMC Cardiovasc Disord. 2022 Jan 7;22:4. doi: 10.1186/s12872-021-02311-z (PMC8742311; doi:10.1186/s12872-021-02311-z)
Supplement: Supplementary file 2 — Additional file 2. Table S2 showed that after mixing medium- risk group and high-risk group in TIMI and GRACE risk groups, the incidence of in-hospital events and long-term outcomes showed no different. Results were showed as value (n). [file 12872_2021_2311_MOESM2_ESM.docx]

**Table S2. In-hospital and long-term outcomes of NSTEMI patients in both medium and high-risk patients.**

|  | GRACE  (medium+high) | TIMI  (medium+high) | P value |
| --- | --- | --- | --- |
| In-hospital events | 51/191, 26.7% | 44/163, 27.0% | 0.951 |
| All-cause mortality | 18/144, 12.5% | 15/121, 12.4% | 0.980 |
| Cardiac mortality | 11/143, 7.7% | 9/120, 7.5% | 0.953 |

Results are showed as value (n).
