# Supplementary material for: Comparison of GRACE and TIMI risk scores in the prediction of in-hospital and long-term outcomes among East Asian non-ST-elevation myocardial infarction patients
Source: BMC Cardiovasc Disord. 2022 Jan 7;22:4. doi: 10.1186/s12872-021-02311-z (PMC8742311; doi:10.1186/s12872-021-02311-z)
Supplement: Supplementary file 3 — Additional file 3. Table S3 showed the difference of baseline characteristics between patients with a high GRACE score and those with a low GRACE score in the TIMI medium-risk group. Results were showed as median (interquartile range), or percentage (n%). [file 12872_2021_2311_MOESM3_ESM.docx]

**Table S3. Baseline difference between GRACE>=140 patients and GRACE<140 patients in TIMI medium risk group.**

| Variables | High-GRACE-score patients | Low-GRACE-score patients | P value |
| --- | --- | --- | --- |
| **On admission** |  |  |  |
| Age (years) | 75.0 (69.0-79.8) | 64.5 (56.0-68.0) | <0.001 |
| DBP (mmHg) | 75.0 (68.0-80.0) | 80.0 (75.0-90.0) | 0.001 |
| SBP (mmHg) | 135.0 (125.0-148.5) | 146.0 (132.5-160.3) | 0.001 |
| Hb (g/L) | 124.0 (110.3-136.8) | 142.0 (129.5-151.5) | <0.001 |
| PLT (*10^9^/L) | 192.5 (156.3-227.5) | 213.0 (176.0-266.5) | 0.022 |
| ALT (U/L) | 20.0 (12.3-27.2) | 25.5 (17.8-37.0) | 0.006 |
| SCr (μmmol/L) | 86.5 (68.5-110.5) | 68.0 (57.5-83.0) | <0.001 |
| TC (mmol/L) | 4.4 (3.5-5.1) | 4.8 (4.1-5.6) | 0.047 |
| LVEF (%) | 58.0 (50.0-64.0) | 61.9 (8.5) | 0.001 |
| CRP (mg/L) | 4.9 (1.1-18.0) | 1.9 (0.8-5.0) | 0.002 |
| BNP (pg/mL) | 268.5 (96.3-437.8) | 76.0 (32.8-213.0) | <0.001 |
| TG (mmol/L) | 1.3 (0.9-2.0) | 1.7 (1.3-2.4) | 0.004 |
| **Previous history (%)** |  |  |  |
| Heart Failure | 9.2 | 0.0 | 0.015 |
| Ischemic stroke | 21.1 | 9.1 | 0.049 |
| Prior MI | 17.1 | 16.7 | 0.945 |
| Angina | 42.1 | 36.4 | 0.485 |
| PCI | 15.8 | 18.2 | 0.704 |
| Hypertension | 67.1 | 72.7 | 0.467 |
| Hyperlipidemia | 6.6 | 3.1 | 0.452 |
| Diabetes | 38.2 | 37.9 | 0.973 |
| Smoking | 26.3 | 45.5 | 0.017 |
| Renal insufficiency | 15.8 | 7.6 | 0.133 |

Results are showed as median (interquartile range), or percentage (n%).
